# Nutrient and popping characteristics of Wyoming‐grown Peruvian popping beans

**DOI:** 10.1002/fsn3.3014

**Published:** 2022-08-09

**Authors:** Justin Bolak, Cody Gifford, Dan Rule, Jim Heitholt, Jill F. Keith

**Affiliations:** ^1^ Department of Family and Consumer Sciences University of Wyoming Laramie Wyoming USA; ^2^ Department of Animal Science University of Wyoming Laramie Wyoming USA; ^3^ Powell Research & Extension Center University of Wyoming Powell Wyoming USA

**Keywords:** dietary fiber, dry beans, legumes, popping beans

## Abstract

American consumers fall short of dietary fiber intake recommended by dietary guidelines. Beans provide protein and fiber, however, less than 14% of adults include them in their daily diets. Nuña beans (*Phaseolus vulgaris* L.), a class of common beans originated in South America and cultivated for growth in North America, possess a unique set of characteristics including flavor profile, popping ability, and nutrient content that may appeal to consumers. The purpose of this study was to evaluate a unique line of Wyoming‐grown popping beans to (1) determine nutrient characteristics and (2) assess popping percentage and shelf stability. Crude protein content was determined for five lines grown in Wyoming utilizing the Dumas method for nitrogen quantification. Total fatty acid content and a fatty acid profile for one line (CO49957) was determined by gas–liquid chromatography. Popping percentage was assessed by heating beans in canola oil in a cast iron pan to induce popping. Storage duration impact on popping was evaluated on CO49957 at 6, 12, and 15 months after harvest. Crude protein content was significantly different between all five lines. Total fatty acid content of CO49957 averaged 2.90 g/100 g wet weight. Average fatty acid profile of CO49957 popped in canola oil comprised oleic acid (41.4%), linoleic acid (20.4%), α‐linolenic acid (18.6%), palmitic acid (10.4%), and stearic acid (2.23%). Popping percentage was 90% (baseline), 100% (6 months), 87% (12 months), and 80% (15 months). Popping beans provide plant‐based protein and fiber while maintaining adequate levels of popping percentage with prolonged storage.

## Graphical abstract

Beans provide protein and fiber, however, few U.S. adults include them in their daily diets. Nuña beans (*Phaseolus vulgaris* L.), a class of common beans originated in South America and cultivated for growth in North America, possess unique characteristics including flavor profile, popping ability, and nutrient content that may appeal to consumers. The purpose of this study was to evaluate a unique line of Wyoming‐grown popping beans to (1) determine nutrient characteristics and (2) assess popping percentage and shelf stability.

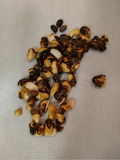

## 1 INTRODUCTION

Dry beans are a significant source of plant‐based protein, dietary fiber, and several micronutrients including folate, zinc, iron, and magnesium (Mitchell et al., 2009). Dietary fiber is of key importance as, on average, U.S. adults consume only 15.6 g of fiber per day (US Department of Agriculture and US Department of Health and Human Services, 2020). This is well below the USDA 2020–2025 Dietary Guidelines for Americans (DGA) daily recommendation of 25 g for women and 38 g for men. Higher fiber intakes have been correlated with normal body weight and decreased postprandial and 24‐h blood glucose levels (Kim et al., 2020; Mitchell et al., 2009). Due to the high fiber and resistant starch content in dry beans, a low glycemic response occurs following consumption relative to other high carbohydrate containing foods (Elazu, 2016; Thompson et al., 2012). Dry beans contain several other non‐nutritive phytochemicals that may play a role in cancer prevention, including phytates, tannins, and saponins (Padhi et al., 2017). Despite the beneficial nutrient aspects of dry beans, individuals of every age category fail to reach the bean and legume consumption recommended in the DGA.

Nuña beans (*Phaseolus vulgaris* L.) are a class of common bean that originated in the Andean region of South America and cultivated for growth in North America by scientists in Colorado and Wisconsin (Pearson et al., 2012). Nuña beans possess a unique popping characteristic. Popping of the nuña bean is a result of pressurized steam being trapped within and between the mesophyll cells and in the cotyledons (Vorwald & Nienhuis, 2009). The pressure culminates as the cotyledon emerges from the seed coat and an audible “pop” is produced. Compared to the conventional U.S. preparation method of dry beans, which includes prolonged soaking followed by sustained boiling, the traditional popping method is more fuel efficient, especially at high altitudes (Vorwald & Nienhuis, 2009). Flavor and texture of popping beans has been described as similar to that of roasted peanuts (Vorwald & Nienhuis, 2009). The unique popping characteristic, preparation method, and flavor profile of popping beans could appeal to consumers.

Dry beans are a significant source of plant‐based protein. Crude protein of pinto beans (22.7%), black beans (24.0%), and red kidney beans (24.0%) has been previously determined (Nosworthy et al., 2017). No identified studies have quantified crude protein percentage of popping beans. As popping beans are derivative of the common bean, *P. vulgaris*, crude protein percentage of popping beans is likely similar to other dry bean species. In addition, fatty acid composition of popping beans is not present in the literature. Fatty acid composition of *P. vulgaris* comprised predominantly the essential fatty acids α‐linolenic acid (39.1%) and linoleic acid (33.7%). Other identified fatty acids include palmitic acid (13.3%), oleic acid (8.2%), and stearic acid (1.9%) (Turco et al., 2016).

Despite the potential benefits and unique characteristics of the nuña popping bean, information specific to popping beans grown in the United States is limited. The purpose of this study was to evaluate Wyoming (WY)‐grown popping beans to (1) determine nutrient characteristics (protein and fatty acid profiles) and (2) assess popping percentage and shelf stability characteristics.

## 2 MATERIALS AND METHODS

### 2.1 Bean samples

**FIGURE 1:**
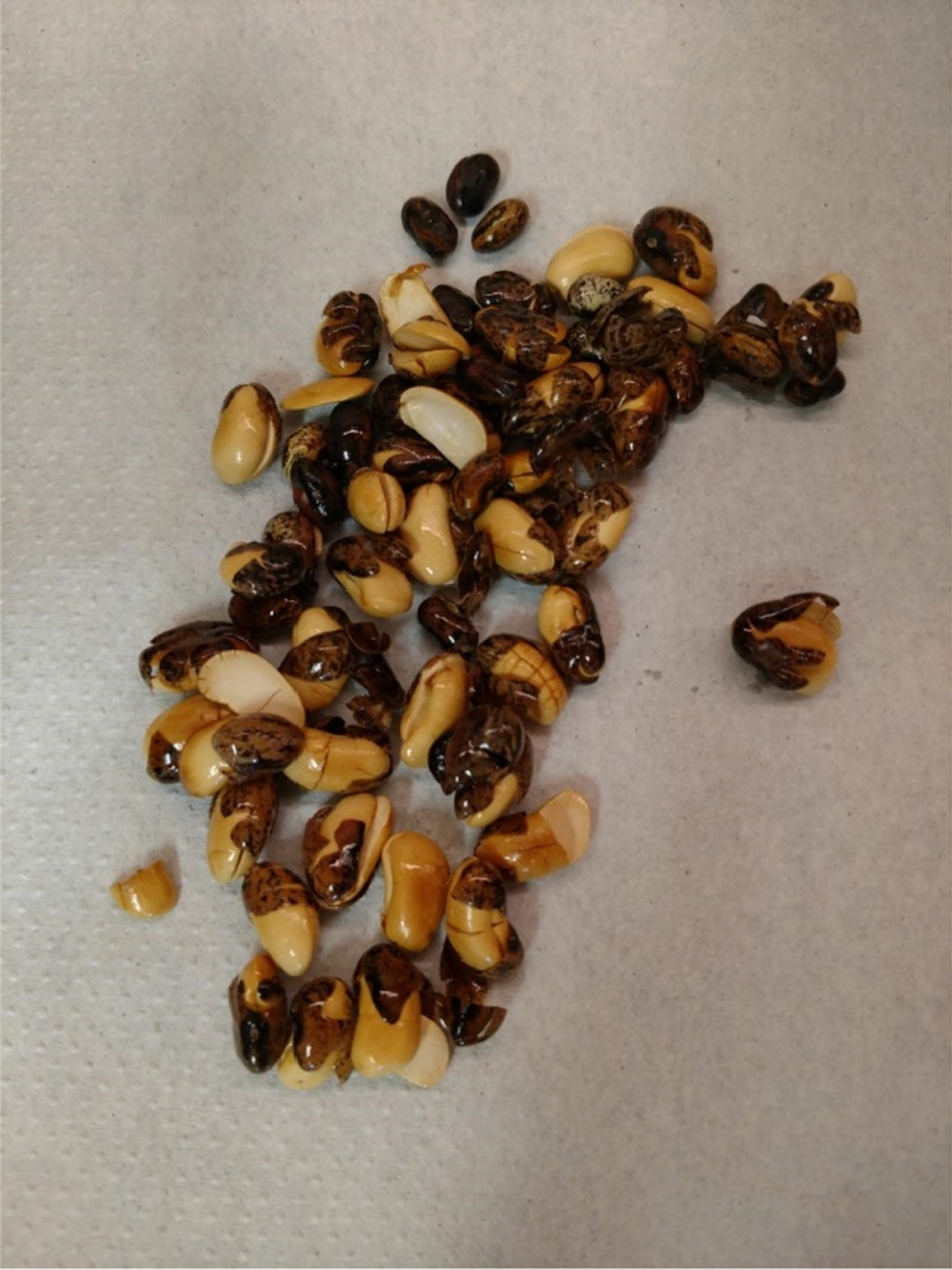
Beans popped in the Human Nutrition and Food laboratory at the University of Wyoming from the CO49957 line.

Popping beans used to determine popping percentage, fatty acid analysis, and crude protein determination (CO49957) were grown at the University of Wyoming's Sustainable Agriculture Research and Extension Center (SAREC) near Lingle, WY. Sample popped beans are displayed in Figure 1. Popping beans were grown under conventional practices, harvested by hand in the fall of 2018 and 2019, and transported to the Human Nutrition and Food (HNF) Laboratory in the Department of Family and Consumer Sciences in the College of Agriculture and Natural Resources at the University of Wyoming (UW). Beans were air dried, hand shelled, and stored in paper bags. Popping beans used exclusively for crude fatty acid analysis (CO49956, PI577678, WI19, WI21) were grown in UW greenhouses in 3 gallon pots containing a mixture of soil, pine bark mix, and sand (1:1:1, v,v,v). Planting date was October 12, 2018. Seeds from CO49956, WI19, and WI21 were harvested 74 days after planting. Seeds from PI577678, a photoperiod sensitive line, were harvested 135 days after planting. Seed shelling and storage followed the procedure described for CO49957.

### 2.2 Popping percentage/shelf stability

Initial testing in the HNF Lab at the UW included heating beans for 1 to 4 min to induce popping using a microwave and air popper (no fat source) and aluminum pan on electric stove and cast iron pan on commercial gas range (using canola oil). Initial testing produced inconsistent popping and quality of beans using the microwave and aluminum pan. Thus, pilot popping testing was conducted on CO49957 using three heating methods to determine finalized method of popping: (1) cast iron skillet on a gas range using canola oil, (2) cast iron skillet on gas range using lard, and (3) air popper. Beans popped with a fat source were heated in a cast iron skillet with one teaspoon (5 ml) of canola oil or lard to induce popping. Beans popped in the air popper (Presto Poplite Hot Air Popcorn Popper) were added to heating chamber for a period of 0.75 to 1.5 min to induce popping. Popping percentages for each method during the pilot testing did not significantly differ. Therefore, the method using canola oil and a cast iron skillet was chosen for the current study as this method was thought to be more representative of oil use in the United States and energy efficient preparation used traditionally with nuña beans. Popping beans (CO49957) were popped in the HNF laboratory. Beans (*n* = 10, *N* = 30) were heated in one teaspoon (5 ml) of canola oil in a cast iron skillet until the oil reached a temperature of 250°C for a period of 1.5 to 2.0 min to induce popping. Temperature was measured using an infrared thermometer (ELECALL EIRT550E) held 12 inches above the surface of the skillet. Popping percentage of line CO49957, from the 2019 harvest, was assessed at baseline (0 months), 6 months, 12 months, and 15 months of storage. Popping percentage was calculated as (popped beans/total beans assessed) × 100.

### 2.3 Fatty acid analysis

Beans popped in canola oil followed the procedure outlined earlier. Excess oil was manually removed with a paper towel from beans popped in oil, and beans were ground to a fine powder using a food processor. Beans popped in the air popper followed the procedure outlined earlier. Air popped beans were then placed directly into the food processor and ground into a fine powder. Samples were partitioned into 0.5 g sample freezer bags, treated with nitrogen gas, sealed, and stored at −80°C until fatty acid analysis.

Fatty acid analysis was conducted by gas–liquid chromatography (GLC) and fatty acid methyl esters were prepared according to the method described by Weston et al. (2008). Briefly, triplicate 0.5 g samples of popping bean were weighed into 16 × 125 mm screw‐top tubes containing 0.5 mg of tridecanoic acid as an internal standard. Fatty acid methyl esters were prepared by adding 4.0 ml of 0.545 N HCl in methanol. Tubes were capped and incubated at 85°C with vortex mixing of each tube for 15 s every 1 min for 1 h. Tubes were then allowed to cool to ambient temperature, and 2.0 ml of H_2_O and 2.0 ml of hexane were added to the tubes, vortex mixed for 15 s, and then centrifuged (Beckman Model TJ‐6 Centrifuge, Beckman Instruments, Inc., Fullerton, CA) at 1300 *g* for 3 min to accelerate phase separation. The upper, hexane phase was transferred to GLC autosampler vials containing a 1‐mm bed of anhydrous sodium sulfate and crimp sealed.

Fatty acid methyl esters were separated using an Agilent 6890 Gas Chromatograph with Agilent 7683 injector (Agilent Technologies, Wilmington, DE) equipped with a flame ionization detector and a 100 m × 0.25 mm i.d. column (SP‐2569, 0.2 μm film thickness, Supelco, Inc. Bellefonte, PA). Oven temperature was maintained at 100°C for 5 min and then increased to 190°C at 10°C min^−1^. Temperature was maintained at 190°C for 10 min and then increased to 220°C at 10°C min^−1^. Temperature was maintained for 23 min. Injector and detector temperatures were 250 and 260°C, respectively. Hydrogen was used as the carrier gas at a split ratio of 25:1 and a constant flow rate of 1.3 ml min^−1^. Fatty acid methyl ester peaks were recorded and integrated using Agilent GC ChemStation software (Agilent Technologies). Individual fatty acid methyl esters were identified by comparing retention times with known fatty acid methyl ester standards (Nu‐Chek Prep., Inc, Elysian, MN).

### 2.4 Crude protein percentage

Crude protein analysis was performed by the WY State Analytical Lab. A LECO TruMacN was used to achieve combustion via the Dumas method for the quantitative determination of nitrogen. The samples (CO49957, CO49956, PI577678, WI19, WI21) were combusted in a furnace composed of “pure” oxygen, at 1100°C, to ensure complete oxidation. The combustion gas was then carried through a series of reagents to remove moisture and carbon dioxide. This allowed for the conversion of nitrogen oxides to molecular nitrogen. These reagents ensured that the sample reaching the detector was nitrogen. The nitrogen was then quantitated using a thermal conductivity (TC) detector. The quantitated nitrogen was then converted to percent crude protein by using the protein conversion factor of 6.25.

### 2.5 Statistical analysis

Analysis of popping percentage compared the difference of baseline (0 months) popping and repeated testing of a time period of 6, 12, and 15 months using repeated measures ANOVA. Analysis of crude protein percentage compared the difference of lines CO49957, CO49956, WI19, WI21, and PI787678 using one‐way ANOVA. Mean value for each line was compared after analysis was repeated in triplicate. Post hoc Tukey's correction was performed to account for family‐wise error. Total fat content and fatty acid composition are reported as the mean value for each nutrient after analysis was repeated in triplicate. Analysis of total fat content and fatty acid profile was accomplished using two‐way ANOVA. Alpha Type I error rate was set at 0.05 for statistical significance. Statistical analyses were performed using Minitab version 18.0 and IBM SPSS version 26.

## 3 RESULTS

### 3.1 Popping percentage

Onset of popping ranged from 230 to 240°C (baseline), 213 to 240°C (6 months), 199 to 231°C (12 months), and 190 to 231°C (15 months). No significant difference in onset of popping temperature between trials was observed. Popping percentage was 90% (baseline), 100% (6 months), 87% (12 months), and 80% (15 months). Popping percentage did not differ significantly at 6, 12, or 15 months as compared to baseline. Popping data are summarized in Table 1.

**TABLE 1:** Shelf stability trial data (mean values) examining effect of storage duration on popping percentage of popping beans

Storage time (months)	Popping (%)	Popping time (min:sec)	Weight (g)	Temp (°C)	*p*
Baseline (0)	90	2:47	4.3	233.3	–
6	100	1:10	5.0	227.7	NS
12	87	3:20	4.5	228.3	NS
15	80	2:30	4.3	221.7	NS

*Note*: NS indicates nonsignificant at *p* ≤ .05.

### 3.2 Fatty acid profile

No significant difference was found when comparing the fatty acid profile of CO49957 harvest year 2018 and 2019 from either air‐popped or oil‐popped beans. Therefore, harvest years were averaged and expressed as either air‐popped or oil‐popped in Tables 2 and 3.

**TABLE 2:** Fatty acid composition (area %) of CO49957 popping beans harvest year 2018 and 2019 air popped or popped in oil along with reference canola oil

Fatty acid^a^	Air popped	Oil popped	Canola oil
C16:0 (palmitic acid)	19.5	10.4	4.86
C18:0 (stearic acid)	2.61	2.23	1.54
C18:1 Δ9 (oleic acid)	10.9	41.4	59.1
C18:2 Δ9,12 (linoleic acid)	24.9	20.4	19.9
C18:3 Δ9,12,15 (α‐linolenic acid)	38.0	18.6	9.15
Σ Saturated^b^	22.1	12.6	6.4
Monounsaturated	10.9	41.4	59.1
Σ Polyunsaturated^c^	62.9	39.0	29.1

^a^ The first number indicates the chain length of the fatty acid and the second number indicates the number of carbon–carbon double bonds (all *cis*) with Δ signifying the location of the double bond(s).

^b^ Σ Saturated = C16:0 + C18:0.

^c^ Σ Polyunsaturated = C18:2 + C18:3.

**TABLE 3:** Fatty acid composition (mg/100 g bean) of CO49957 air popped or popped in oil

Fatty acid^a^	Air popped^a^	Oil popped
C16:0 (palmitic acid)	262	313
C18:0 (stearic acid)	35	65***
C18:1 Δ9 (oleic acid)	147	1199***
C18:2 Δ9,12 (linoleic acid)	335	591***
C18:3 Δ9,12,15 (α‐linolenic acid)	514	541
Σ Saturated^b^	297	378*
Monounsaturated	147	1199***
Σ Polyunsaturated^c^	849	1131***
Σ FA/100 g^d^	1348	2903***

*Note*: Values are rounded to the nearest whole number.

^a^ The first number indicates the chain length of the fatty acid and the second number indicates the number of carbon–carbon double bonds (all *cis*) with Δ signifying the location of the double bond(s).

^b^ Σ Saturated = C16:0 + C18:0.

^c^ Σ Polyunsaturated = C18:2 + C18:3.

^d^ Σ FA/100 g = total fatty acids per 100 grams.

* *p* < .05 as compared to air popped.

*** *p* < .001 as compared to air popped.

Fatty acid profile is detailed in Table 2 and is expressed as area % (mg of fatty acid/100 mg of total fatty acids × 100) of the entire gas–liquid chromatography profile. The primary fatty acids found in air‐popped CO49957 were α‐linolenic acid (C18:3, 38.0%) and linoleic acid (C18:2, 24.9%), with palmitic acid (C16:0, 19.5%), oleic acid (C18:1, 10.9%), and stearic acid (C18:0, 2.61%) also detected in significant quantities. The fatty acid profile of air‐popped beans consisted of polyunsaturated fatty acids (62.9%), saturated fatty acids (22.1%), and monounsaturated fatty acids (10.9%).

Table 3 details the amount of each fatty acid per 100 g of bean sample. Preparation of popping beans in canola oil, as compared to air popping, significantly increased stearic acid (86%, *p* < .001), oleic acid (715%, *p* < .001), and linoleic acid content (76%, *p* < .001). An increase in palmitic acid (19%) and α‐linolenic acid (5%) was observed but did not reach statistical significance. A significant increase (115%, *p* < .001) in total fatty acid content was observed when popping beans in canola oil as compared to air popping. Air popped beans contained 1348 mg/100 g bean of total fat. Oil popped beans contained 2903 mg/100 g bean of total fat. Popping beans in canola oil as compared to air popping also significantly increased monounsaturated fat content (715%, *p* < .001), polyunsaturated fat content (33%, *p* < .001), and saturated fat content (27%, *p* = .022).

### 3.3 Crude protein percentage

Crude protein content for popping bean lines was 20.9% (CO49957), 17.3% (CO49956), 18.9% (PI78), 22.1% (WI19), and 20.1% (WI21). Crude protein differed significantly (*p* < .001) between all lines.

## 4 DISCUSSION

Popping percentage was not significantly reduced at any time point as compared to immediately postharvest. An increase in popping percentage was observed at 6 months (100%) as compared to immediately postharvest (90%). Although seed moisture was not determined in this study, the increase in popping percentage could be explained by a decrease in moisture content as the beans aged. Vorwald and Nienhuis (2009) showed that nuña beans with a seed moisture content below 5% maximized popping percentage. A numeric decrease in popping percentage was observed at 12 and 15 months, but was not significant. Other researchers identified popping percentage between 17% and 81% within 3 months of harvest, but were not evaluated for popping ability after 3 months (Pearson et al., 2012). Future research should extend the duration of shelf storage to determine if a significant decline in popping percentage is observed beyond 15 months of storage time. Onset of popping averaged 233°C (baseline), 228°C (6 months), 228°C (12 months), and 221°C (15 months). These results are slightly lower than those reported by Vorwald and Nienhuis (2009), who showed that ideal popping temperature was above 244°C. The difference in popping temperature could be explained by differences in popping modality. Vorwald and Nienhuis (2009) used an air popper that heats beans through convection. In this study, beans were heated through conduction. Popping trials in this study were conducted at an altitude of 7220 feet. Vorwald and Nienhuis (2009) conducted popping testing at an altitude of approximately 875 feet. Decreased atmospheric pressure, as a result of high altitude, could have affected steam pressurization within the cotyledon. This is another possible explanation for the disparity in the ideal popping temperature published by Vorwald and Nienhuis (2009) and the popping temperatures found in the current study. For use in home or commercial settings, consistent popping without burning or overcooking would be an important factor in standardizing popping method.

The quick cooking method used in this study has the advantage of decreased preparation time and fuel usage. Common dry bean preparation in the United States involves long soaking and boiling times. The benefits from decreased preparation time and fuel requirements are magnified in the traditional popping bean preparation environments in the highlands of Peru. In boiling scenarios, the decreased boiling point that is characteristic of high elevation, extends cooking time beyond what would be experienced at sea level. Increased cooking time requires collecting additional vegetation as fuel, and local vegetation is often a scarce commodity in the highlands of Peru (Zimmerer, 1992). While consumers in the highlands of Peru benefit from the popping bean's decreased fuel demand, consumers in the United States could benefit from decreased cooking time by using less propane, natural gas, or electricity for cooking.

The fatty acid profile and total fat content of popping beans were similar to the fatty acid profile of other common bean varieties determined by Turco et al. (2016). Comparison of fatty acid profiles are shown in Table 4.

**TABLE 4:** Fatty acid composition (% of total profile) and total fat content of air popped popping bean, commercial black bean, and commercial kidney bean

Fatty acid^a^	Popping bean	Black bean	Kidney bean
C16:0	19.5	10.7	12.3
C:18:0	2.61	1.8	1.4
C18:1 Δ9	10.9	9.3	9.5
C18:2 Δ9,12	24.9	31.1	24.1
C18:3 Δ9,12,15	38.0	41.7	46.0
Total fat/100 g	1.35	1.30	2.20

^a^ The first number indicates the chain length of the fatty acid and the second number indicates the number of double bonds (all *cis*) with Δ signifying the location of the double bond(s).

Although there were no statistically significant differences in fatty acid profile when comparing harvest year 2018 to harvest year 2019, variability of fatty acid content can be influenced by environmental factors such as temperature during growth stages, planting time, rainfall, drought, and harvest time (Saad Bin Mustafa et al., 2016). The combined average of air popped CO49957, for both harvest years 2018 and 2019, shows the majority of the popping bean's fatty acid profile comprised α‐linolenic acid (38.0%) and linoleic acid (24.9%). Linoleic acid, an omega‐6 fatty acid, and α‐linolenic acid, an omega‐3 fatty acid, are both essential fatty acids that must be obtained from the diet as biological synthesis is not possible in humans. High dietary intakes of linoleic acid have been associated with diminished cardiovascular disease risk, improved glycemic control, decreased insulin resistance, and decreased risk of developing type 2 diabetes (Blondeau et al., 2015). α‐Linolenic acid has been shown to have neuroprotective, anti‐inflammatory, and antidepressant properties and is required for normal brain development and function (Blondeau et al., 2015). Although the total fat content of popping beans was relatively low, inclusion of popping beans in a healthy diet may contribute to the aforementioned health attributes.

Fatty acid profile detailed in Table 3 is expressed in mg of fatty acid per 100 g of popping beans. A significant increase in total fatty acid content was observed when popping beans in canola oil as compared to air popping for both harvest year 2018 (1548 vs. 3065 mg/100 g bean) and harvest year 2019 (1148 vs. 2439 mg/100 g bean). For harvest year 2018, popping in oil as compared to air popping significantly increased palmitic acid (16%), stearic acid (87%), oleic acid (686%), and linoleic acid (57%). For harvest year 2019, popping in oil as compared to air popping significantly increased stearic acid (88%), oleic acid (784%), and linoleic acid (107%). The large increase in oleic acid content was observed primarily due to the amount of oleic acid (59%) in canola oil. For consumers, this information is valuable to assist with selecting which popping modality or oil to use. Popping beans (100 g) were roughly equivalent to half cup, which could serve as guidance in serving size determination. A half cup serving of air popped beans would contain 1.30 g of total fat. A half cup serving of popping beans prepared in canola oil would contain 2.64 g of total fat. Crude fat content of air popped beans is much lower than crude fat content of air popped popcorn (3.9 g/100 g) as determined by Khan et al. (2016). As popcorn is a popular snack food with a significant commercial demand, low‐fat microwavable options have been developed with a fat content as low as 5.5 g/100 g (Nguyen et al., 2012). Conversely, preparation of stovetop popcorn similar to the methods utilized in this study has resulted in crude fat content as high as 26.0 g/100 g (Khan et al., 2016). Thus, half cup of oil popped beans with 2.90 g of fat is well below current guidance for low‐fat snack options.

Findings from this study indicate that popping beans can provide a significant source of plant‐based protein. Dry beans provide the majority of protein intake in regions of the world where animal protein sources are consumed in lesser amounts, such as sub‐Saharan Africa and the Caribbean. Consumption of dry beans can increase protein intake without concurrent saturated fat intake as would be seen with beef and full‐fat dairy consumption. For example, dry kidney beans contain 0.15 g of saturated fat in 100 g, while whole milk contains 1.86 g/100 g and 80% lean ground beef contains 7.58 g/100 g (USDA Food Data Central). Dry bean consumption has been associated with a protective effect against the development of chronic diseases including a decreased incidence of diabetes, cardiovascular disease, and prostate, colon, and breast cancer (Mitchell et al., 2009). Crude protein content of the five popping bean lines examined in this study ranged from 17.3% to 22.1%. This is similar to the range of crude protein content of black beans (20%–25%) reported by Evangelho et al. (2017) and crude protein content of red kidney beans (23.9%) and pinto beans (22.7%) reported by Nosworthy et al. (2017) and Evangelho et al. (2017). By comparison, crude protein content of popping beans is twice the crude protein content of popcorn (8.1%–10.5%) (Park et al., 2000). A half cup serving of CO49957 popping beans would provide approximately 21 g of protein. Popping beans can offer a significant source of protein for individuals seeking a greater amount of plant‐based foods in their diet, or for individuals living in regions where animal‐based protein sources are scarce or expensive.

One limitation of this study is extrapolation of popping percentage data. As popping trials were conducted with *n* = 10 beans per trial, popping percentages determined in this study may not be indicative of preparation of large‐scale batches of popping beans. Additional research should consider large‐scale popping trials. Another limitation is the length of the shelf stability trial. A decrease in popping percentage was observed at 12 and 15 months of storage, but did not reach statistical significance. Future research should continue shelf stability trials beyond 15 months to determine when the decreased popping percentage reaches statistical significance and if storage techniques influence the observed decrease in popping percentage. Both pieces of information are critical to determining the shelf life of popping beans.

## 5 CONCLUSION/IMPLICATIONS

Despite the nutritional and health benefits associated with regular bean consumption, individuals of every age category fail to reach the DGA recommendations for bean, pea, and lentil consumption (US Department of Agriculture and US Department of Health and Human Services, 2020). The popping bean's unique popping characteristic, paired with its quick preparation time as compared to other dry bean varieties, may appeal to consumers. Regular inclusion of popping beans, as a part of a healthy dietary pattern, can provide a significant source of plant‐based proteins, dietary fiber, and beneficial micronutrients. With individuals of every age category failing to meet the DGA recommendations for dry bean and legume consumption, strategies aimed at increasing consumer consumption of dry beans and education regarding preparation and use in meal planning should be pursued.

## CONFLICT OF INTEREST

The authors declare that they do not have any conflict of interest.

## ETHICS STATEMENT

This study does not involve any human or animal testing.

## Data Availability

The data that support the findings of this study are available from the corresponding author upon reasonable request.

## References

[fsn33014-bib-0001] Blondeau, N. , Lipsky, R. H. , Bourourou, M. , Duncan, M. W. , Gorelick, P. B. , & Marini, A. M. (2015). Alpha‐linolenic acid: An omega‐3 fatty acid with neuroprotective properties ‐ready for use in the stroke clinic? BioMed Research International, 2015, 519830. 10.1155/2015/519830 25789320 PMC4350958

[fsn33014-bib-0002] Elazu, C. O. (2016). The concept of low glycemic index and glycemic load foods as panacea for type 2 diabetes mellitus; prospects, challenges and solutions. African Health Sciences, 16, 468–479. 27605962 10.4314/ahs.v16i2.15 PMC4994556

[fsn33014-bib-0003] Evangelho, J. A. , Vanier, N. L. , Pinto, V. Z. , De Berrios, J. J. , Renato, A. , Dias, G. , & Zavareze, E. R. (2017). Black bean (*Phaseolus vulgaris* L.) protein hydrolysates: Physiochemical and functional properties. Food Chemistry, 214, 460–467. 10.1016/j.foodchem.2016.07.046 27507499

[fsn33014-bib-0004] Khan, M. U. , Majeed, M. , Tayyab, M. , Shariati, M. A. , & Rashidzadeh, S. (2016). Chemical and nutritional properties of some commercial available corn and wheat products. Journal of Microbiology, Biotechnology, and Food Sciences, 6, 863–866. 10.15414/jmbfs.2016.6.2.863-866

[fsn33014-bib-0005] Kim, H. K. , Nanba, T. , Ozaki, M. , Chijiki, H. , Takahashi, M. , Fukazawa, M. , Okubo, J. , & Shibata, S. (2020). Effect of the intake of a snack containing dietary fiber on postprandial glucose levels. Food, 9(10), 1500. 10.3390/foods9101500 PMC7589548 33092177

[fsn33014-bib-0006] Mitchell, D. C. , Lawrence, F. R. , Hartman, T. J. , & Curran, J. M. (2009). Consumption of dry beans, peas, and lentils could improve diet quality in the U.S. population. Journal of the American Dietetic Association, 109(5), 909–912. 10.1016/j.jada.2009.02.029 19394480

[fsn33014-bib-0007] Nguyen, V. , Cooper, L. , Lowndes, J. , Melanson, K. , Angelopoulos, T. J. , Rippe, J. M. , & Reimers, K. (2012). Popcorn is more satiating than potato chips in normal‐weight adults. Nutrition Journal, 11, 71. 10.1186/1475-2891-11-71 22978828 PMC3502142

[fsn33014-bib-0008] Nosworthy, M. G. , Neufeld, J. , Frohlich, P. , Young, G. , Malcolmson, L. , & House, J. D. (2017). Determination of the protein quality of cooked Canadian pulses. Food Science and Nutrition, 5, 896–903. 10.1002/fsn3.473 28748078 PMC5521049

[fsn33014-bib-0009] Padhi, E. M. T. , Liu, R. , Hernandez, M. , Tsao, R. , & Ramdath, D. D. (2017). Total polyphenol content, carotenoid, tocopherol and fatty acid composition of commonly consumed Canadian pulses and their contribution to antioxidant activity. Journal of Functional Foods, 38, 602–611. 10.1016/j.jff.2016.11.006

[fsn33014-bib-0010] Park, D. , Allen, K. G. D. , Stermitz, F. R. , & Maga, J. A. (2000). Chemical composition and physical characteristics of unpopped popcorn hybrids. Journal of Food Composition and Analysis, 13, 921–934. 10.1006/jfca.2000.0943

[fsn33014-bib-0011] Pearson, C. H. , Ogg, J. B. , Brick, M. A. , & Berrada, A. (2012). Popping and yield characteristics of nuna bean lines developed for temperate climates. Agronomy Journal, 104, 1574–1578.

[fsn33014-bib-0100] Saad Bin Mustafa, H. , Hasan, E. , Sarwar, S. , Qayyum, A. , & Mahmood, T. (2016). Influence of climatic conditions on chemical configuration of seeds in safflower, soybean, linseed and sesame. Nature and Science, 14, 125–140. 10.7537/marsnsj140916.18

[fsn33014-bib-0012] Thompson, S. V. , Winham, D. M. , & Hutchins, A. M. (2012). Bean and rice meals reduce postprandial glycemic response in adults with type 2 diabetes: A cross‐over study. Nutrition Journal, 11, 23. 10.1186/1475-2891-11-23 22494488 PMC3489574

[fsn33014-bib-0013] Turco, V. L. , Potorti, A. G. , Rando, R. , Ravenda, P. , Dugo, G. , & Di Bella, G. (2016). Functional properties and fatty acid profile of different beans varieties. Natural Product Research, 30(19), 2243–2248. 10.1080/14786419.2016.1154056 26949141

[fsn33014-bib-0014] US Department of Agriculture and US Department of Health and Human Services . (2020). Dietary guidelines for Americans, 2020‐2025 (9th ed.). https://www.dietaryguidelines.gov/sites/default/files/2020‐12/Dietary_Guidelines_for_Americans_2020‐2025.pdf

[fsn33014-bib-0015] Vorwald, J. , & Nienhuis, J. (2009). Effects of seed moisture content, cooking time, and chamber temperature on nuña bean (*Phaseolus vulgaris* L.) popping. HortScience, 44, 135–137.

[fsn33014-bib-0016] Weston, T. R. , Derner, J. D. , Murrieta, C. M. , Rule, D. C. , & Hess, B. W. (2008). Comparison of catalysts for direct transesterificiation of fatty acids in freeze‐dried forage samples. Crop Science, 48, 1636–1641. 10.2135/cropsci2007.07.0376sc

[fsn33014-bib-0017] Zimmerer, K. S. (1992). Biological diversity and local development: “Popping beans” in the Central Andes. Mountain Research and Development, 12, 47–61. 10.2307/3673747

